# The Behaviour of IL-6 and Its Soluble Receptor Complex during Different Waves of the COVID-19 Pandemic

**DOI:** 10.3390/life14070814

**Published:** 2024-06-27

**Authors:** Gaetano Di Spigna, Bianca Covelli, Maria Vargas, Roberta Di Caprio, Valentina Rubino, Carmine Iacovazzo, Filomena Napolitano, Giuseppe Servillo, Loredana Postiglione

**Affiliations:** 1 Department of Translational Medical Sciences, University of Naples “Federico II”, 80131 Naples, Italy; gaetano.dispigna@unina.it (G.D.S.); bcovelli@unina.it (B.C.); rob.dicaprio@gmail.com (R.D.C.); valentina.rubino@unina.it (V.R.); filomena.napolitano@unina.it (F.N.); 2 Department of Neurosciences, Reproductive and Odontostomatological Sciences, University of Naples “Federico II”, 80131 Naples, Italy; vargas.maria82@gmail.com (M.V.); carmine.iacovazzo@unina.it (C.I.); servillo@unina.it (G.S.)

**Keywords:** COVID-19, severe acute respiratory syndrome corona virus 2, IL6/IL6 receptor complex

## Abstract

In late December 2019, SARS-CoV-2 was identified as the cause of a new pneumonia (COVID-19), leading to a global pandemic declared by the WHO on 11 March 2020, with significant human, economic, and social costs. Although most COVID-19 cases are asymptomatic or mild, 14% progress to severe disease, and 5% develop critical illness with complications such as interstitial pneumonia, acute respiratory distress syndrome (ARDS), and multiple organ dysfunction syndrome (MODS). SARS-CoV-2 primarily targets the respiratory system but can affect multiple organs due to the widespread presence of angiotensin-converting enzyme 2 (ACE2) receptors, which the virus uses to enter cells. This broad distribution of ACE2 receptors means that SARS-CoV-2 infection can lead to cardiovascular, gastrointestinal, renal, hepatic, central nervous system, and ocular damage. The virus triggers the innate and adaptive immune systems, resulting in a massive cytokine release, known as a “cytokine storm”, which is linked to tissue damage and poor outcomes in severe lung disease. Interleukin-6 (IL-6) is particularly important in this cytokine release, with elevated levels serving as a marker of severe COVID-19. IL-6 is a multifunctional cytokine with both anti-inflammatory and pro-inflammatory properties, acting through two main pathways: classical signalling and trans-signalling. Classical signalling involves IL-6 binding to its membrane-bound receptor IL-6R and then to the gp130 protein, while trans-signalling occurs when IL-6 binds to the soluble form of IL-6R (sIL-6R) and then to membrane-bound gp130 on cells that do not express IL-6R. The soluble form of gp130 (sgp130) can inhibit IL-6 trans-signalling by binding to sIL-6R, thereby preventing it from interacting with membrane-bound gp130. Given the central role of IL-6 in COVID-19 inflammation and its association with severe disease, we aimed to analyse the behaviour of IL-6 and its soluble receptor complex during different waves of the pandemic. This analysis could help determine whether IL-6 levels can serve as prognostic markers of disease severity.

## 1. Introduction

In late December 2019, a novel RNA beta-coronavirus was identified as the causative agent of pneumonia cases of unknown origin. This virus was subsequently named Severe Acute Respiratory Syndrome Coronavirus 2 (SARS-CoV-2), and the respiratory illness it causes was termed Coronavirus Disease 2019 (COVID-19) [1,2]. The global spread of SARS-CoV-2 and the substantial death toll from COVID-19 led the World Health Organization (WHO) to declare a pandemic on 11 March 2020 [1,2]. To date, the pandemic has exacted a high toll in terms of human lives lost, economic repercussions, and increased poverty.

While most COVID-19 patients are asymptomatic or experience mild, self-limiting disease, 14% develop severe disease, and 5% progress to critical illness with complications such as interstitial pneumonia, respiratory failure with acute respiratory distress syndrome (ARDS), and multiple organ dysfunction syndrome (MODS) [3]. It is well-established that SARS-CoV-2 primarily affects the respiratory system, although other organs are also involved [4]. The virus enters host cells by binding its spike-like surface projections to the angiotensin-converting enzyme 2 (ACE2) receptor, which is mainly expressed on alveolar epithelial cells and in varying degrees across nearly all human organs. Thus, although the primary target of SARS-CoV-2 infection is the lung, the widespread distribution of ACE2 receptors in other organs can lead to cardiovascular, gastrointestinal, kidney, liver, central nervous system, and ocular damage, which must be closely monitored during the course of the disease [5,6].

Towards the end of the second wave, when high vaccination rates among the adult Italian population had raised hopes of a return to pre-pandemic life, a newly identified variant of SARS-CoV-2, named the Delta variant, led to a resurgence in COVID-19 infections [7]. Several variants of SARS-CoV-2 have emerged and spread globally during the COVID-19 pandemic. Based on evidence of increased transmissibility, disease severity, and reduced therapeutic effectiveness, the Centers for Disease Control and Prevention (CDC) have defined three different levels of threat associated with variants. These include variants of interest (VOI), such as Iota (B.1.526), Eta (B.1.525), Kappa (B.1.617.1), B.1.617.3, Epsilon (B.1.427/B.1.429), Lambda (C.37), and Zeta (P.2); variants of concern (VOC), including Alpha (B.1.1.7), Beta (B.1.351), Gamma (P.1), Delta (B.1.617.2); and variants of high consequence [8].

The virus activates both the innate and adaptive immune systems, resulting in the release of numerous cytokines, known as a “cytokine storm”, which is directly correlated with tissue injury and poor prognosis in cases of severe lung disease [9]. In particular, interleukin (IL)-6 seems to play an important role in driving this cytokine release, and thus, increased levels have been recognised as a hallmark inflammatory signature in the sera of COVID-19 patients [10]. IL-6 is a polyfunctional cytokine with both anti-inflammatory and pro-inflammatory properties that exerts its several functions through two main signal transduction pathways [11]. In the classical signalling pathway, IL-6 binds to the membrane-bound IL-6 receptor (IL-6R) to form a complex that then binds to the membrane glycoprotein gp130, inducing its homo-dimerization and initiating intracellular signalling. This type of signalling is limited to cells that express IL-6R on their surface, such as hepatocytes, macrophages, neutrophils, and T cells [12]. 

Cells that do not express IL-6R, such as stromal and epithelial cells, can still respond to IL-6 through the soluble form of the receptor (sIL-6R). sIL-6R binds to IL-6 in circulation and then anchors to the membrane-bound gp130 receptor, initiating the trans-signalling pathway. Therefore, sIL-6R increases IL-6 function and is considered the agonistic form of its receptor. When IL-6 levels increase, its effects are widely expressed because gp130 molecules are ubiquitous and present even in cells lacking membrane-bound IL-6R. Conversely, a soluble form of glycoprotein 130 (sgp130) can form a complex with sIL-6R, preventing it from binding to membrane-bound gp130. In this case, sgp130 decreases IL-6 function and acts as the antagonistic form of the IL-6 receptor system [13].

Given the central role of IL-6 in the inflammatory pathology of COVID-19 and the consistent association between elevated IL-6 levels and severe disease outcomes observed early in the pandemic, we aim to analyse IL-6 and its soluble receptor complex behaviour across different waves of the COVID-19 pandemic. This analysis may reveal differences among the waves and help determine if these markers can be used as prognostic indicators of disease severity.

## 2. Materials and Methods

### 2.1. Study Design and Population

In this prospective observational study, patients with confirmed SARS-CoV-2 infection admitted to the intensive care unit (ICU) of the University of Naples Federico II during different COVID-19 waves were enrolled. Specifically, patient recruitment spanned from 10 March 2020 to 31 October 2021 and included the following cohorts: (i) N = 23, enrolled between 10 March and 30 April 2020 (WAVE I); (ii) N = 104, enrolled between 12 September and 31 December 2020 (WAVE II); and (iii) N = 30, enrolled between 7 September and 31 October 2021 (WAVE III) [14,15,16,17]. 

Additionally, a group of patients (N = 20) admitted to the ICU and infected with the Delta variant of SARS-CoV-2 was included in the study.

According to WHO interim guidance, a confirmed case of COVID-19 was defined by a positive real-time reverse transcription polymerase chain reaction (RT-PCR) assay result for SARS-CoV-2 from a nasopharyngeal swab sample [18]. Healthy volunteers matched by age and sex (N = 32 in WAVE I, N = 52 in WAVE II, and N = 28 in WAVE III) were enrolled as controls.

The Ethics Committee (Azienda Ospedaliera Universitaria “Federico II”—Naples, 2022, protocol number: 155/120) approved the investigative protocol.

The experimental protocol adhered to the current version of the Declaration of Helsinki, and each subject provided written informed consent before participating in the study.

### 2.2. Biological Samples

Fasting venous blood samples were collected from all patients and healthy subjects. Sera were obtained through standard centrifugation, divided into aliquots, and stored frozen until analysis. Samples were thawed only once and immediately assayed to ensure the integrity of the specimens. Cytokine evaluations were performed using a small amount of sera samples collected at patient admission to perform routine biochemical analysis.

### 2.3. Quantitative Determination of IL-6, sIL-6R and sgp130

A quantitative determination of IL-6 serum levels was performed using Chemiluminescent Immunoassay (CLIA) on the IMMULITE 2000 system (Siemens Healthcare Diagnostics, Milano, Italy). Conversely, serum levels of sIL-6R and sgp130 were measured using an automated Enzyme-Linked Immunosorbent Assay (ELISA) on the Triturus System analyser (Grifols, Vicopisano, Italy). R&D Quantikine ELISA Kits (R&D Systems, Minneapolis, MN, USA, distributed by DIACHEM S.r.l., Giugliano in Campania, Italy) were employed for all determinations. The intra- and inter-assay coefficients of variation were less than 5% for IL-6 and sgp130 serum levels and less than 10% for sIL-6R evaluation. Reference values were assessed in healthy control subjects with no family history of COVID-19, recruited from the employees of Azienda Ospedaliera Universitaria “Federico II”, Naples, Italy.

#### 2.3.1. Variables

Demographic, clinical, and analytical data were recorded for each patient to describe the clinical phenotype. The analytical data included leukocytes, lymphocytes, neutrophils, platelets, bilirubin, creatinine, glucose, troponin Ths, C-reactive protein (CRP), lactate dehydrogenase (LDH), ferritin, procalcitonin, and D-dimer levels.

#### 2.3.2. cDNA Synthesis and Amplicon Libraries

RNA was extracted from nasopharyngeal swabs of patients using the QIAamp Viral RNA Mini Kit (Qiagen, Hilden, Germany) following the manufacturer’s instructions. The concentration and quality of all extracted RNA samples were measured and verified using Nanodrop 2000 (Thermo Fisher Scientific, Waltham, MA, USA). Viral genomes were amplified using a multiplex approach with version 1 of the CleanPlex SARS-CoV-2 Research and Surveillance Panel (Paragon Genomics, Hayward, CA, USA), starting with 50 ng of total RNA, followed by Illumina sequencing on a NextSeq 500 (Illumina, San Diego, CA, USA).

The generated libraries were controlled with a High-Sensitivity Labchip and quantified using the Qubit Fluorometric Quantitation system (Thermo Fisher Scientific, Waltham, MA, USA). Raw data were trimmed and analysed using CLC Workbench 5 bioinformatics software and the Basic Local Alignment Search Tool (BLAST). Italian sequences imported into the GenBank database (https://www.ncbi.nlm.nih.gov/genbank, 1 April 2024) from March 2020 to November 2021, with released accession numbers, were used to construct phylogenetic trees.

#### 2.3.3. Phylogenetic Analysis

Sequences for the designated variants of concern and variants of interest were downloaded from GISAID (Global Initiative on Sharing All Influenza Data). Multiple sequence alignment (MSA) was performed using MEGA X software, and phylogenetic trees were constructed using the 1000 replicate bootstrap method.

#### 2.3.4. Statistical Analysis

Data that passed the normality test were analyzed using a two-tailed *t*-test. If the data did not pass the normality test, the Mann–Whitney test was employed to calculate statistical differences. Statistical analyses were conducted using GraphPad Prism 6.0 (GraphPad Software Inc., La Jolla, CA, USA) or SAS software v 9.3 (SAS Institute Inc., Cary, NC, USA). A *p*-value less than 0.05 was considered statistically significant.

## 3. Results

### 3.1. Patient’s’ Characteristics

A total of 181 patients were enrolled, including 27 ICU patients hospitalised for COVID-19 during WAVE I, 104 during WAVE II, and 30 during WAVE III. The mean ± SD age of patients was 66.88 ± 14.48 years, with 78.26% males in WAVE I; 68.2 ± 18.32 years, with 65.38% males in WAVE II; and 67.42 ± 14.86 years, with 76.67% males in WAVE III. Hypertension, diabetes, and obesity were the main comorbidities. The mean time from the first positive swab to ICU admission was 5 days, while the length of ICU stay ranged from 15 to 20 days in WAVE I, 15 to 10 days in WAVE II, and 7 to 10 days in WAVE III. Demographic and clinical characteristics of patients are summarised in Table 1.

**Figure 1:**
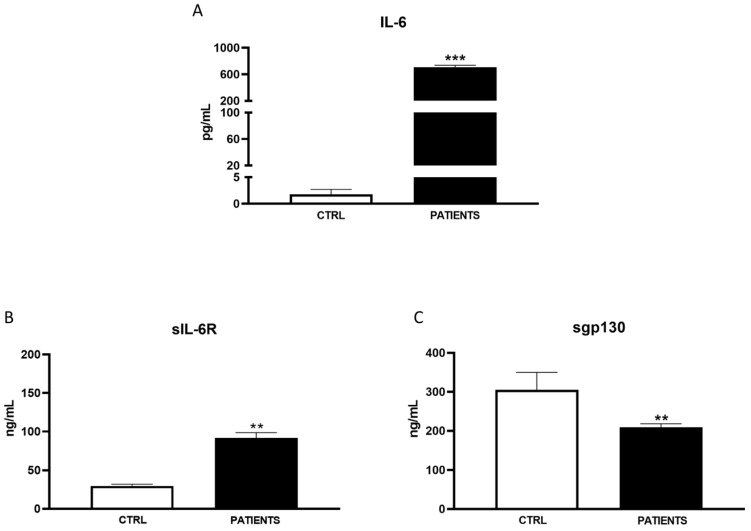
Analysis of IL-6 (pg/mL) (**A**), sIL-6R (ng/mL) (**B**), and sgp130 levels (ng/mL) (**C**) in WAVE I patients. Data are displayed as mean ± SD. Statistical analysis was performed with Mann–Whitney test with respect to Ctrl. ** *p* < 0.01, *** *p* < 0.001.

**Figure 2:**
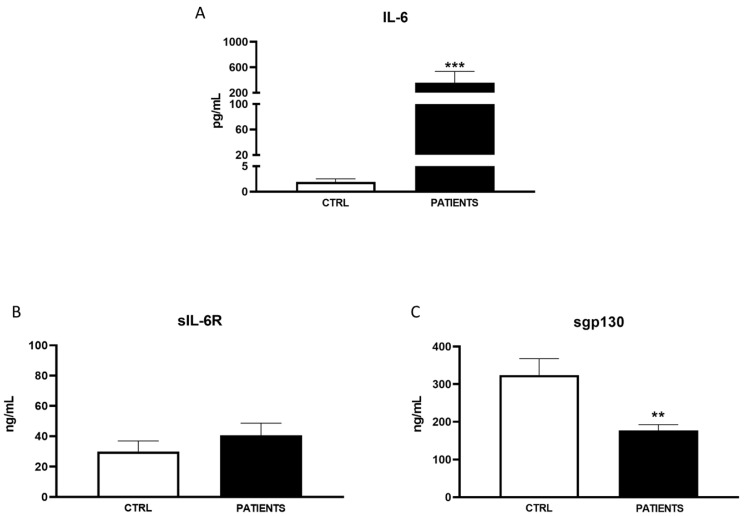
Analysis of IL-6 (pg/mL) (**A**), sIL-6R (ng/mL) (**B**), and sgp130 levels (ng/mL) (**C**) in WAVE II patients. Data are displayed as mean ± SD. Statistical analysis was performed with Mann–Whitney test with respect to Ctrl. ** *p* < 0.01, *** *p* < 0.001.

**Figure 3:**
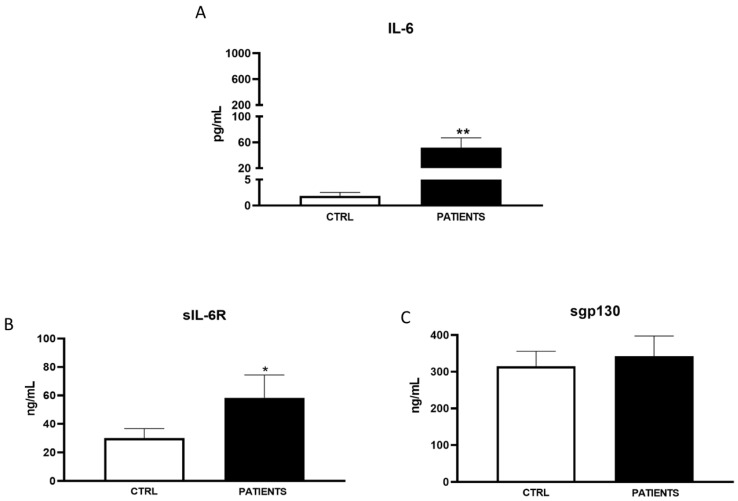
Analysis of IL-6 (pg/mL) (**A**), sIL-6R (ng/mL) (**B**), and sgp130 levels (ng/mL) (**C**) in WAVE III patients. Data are displayed as mean ± SD. Statistical analysis was performed with Mann–Whitney test with respect to Ctrl. * *p* < 0.05, ** *p* < 0.01.

In WAVE I, serum levels of IL-6 significantly increased in COVID-19 patients compared to healthy controls (702.99 ± 1932.54 pg/mL vs. 1.81 ± 0.89 pg/mL, *p* < 0.001). Particularly, the amount of IL-6 in patients was approximately 400 times higher than in healthy subjects (Figure 1A). A similar trend was observed for the IL-6 agonist sIL-6R. It had an amount almost triple in infected patients compared to controls (90.42 ± 74.29 ng/mL vs. 29.91 ± 8.16 ng/mL, *p* < 0.01) (Figure 1B). Conversely, sgp130 significantly decreased in patients with respect to controls (217.92 ± 53.14 ng/mL vs. 305.24 ± 44.99 ng/mL, *p* < 0.01) during the first phase of infection (Figure 1C). In WAVE II, IL-6 levels were significantly higher in patients than in the controls, even if to a minor extent with respect to WAVE I (265.51 ± 976.82 pg/mL vs. 1.92 ± 0.58 pg/mL, *p* < 0.001) (Figure 2A). Moreover, sIL-6R weakly increased with respect to healthy subjects (39.71 ± 17.87 ng/mL vs. 30.01 ± 7.68 ng/mL) (Figure 2B). A greater decrease in sgp130 levels (181.52 ± 67.29 ng/mL) compared to controls (324.31 ± 43.58 ng/mL) was found (*p* < 0.01) (Figure 2C). In WAVE III, the serum levels of IL-6 and sIL-6R continued to be significantly increased in COVID-19 patients compared to healthy controls (63.15 ± 18.5 pg/mL vs. 1.91 ± 0.89 pg/mL, *p* < 0.01 and 75.21 ± 14.29 ng/mL vs. 26.51 ± 8.16 ng/mL, *p* < 0.05, respectively). However, there was a substantial decrease in IL-6 values with respect to the previous waves. In particular, the amount of the cytokine in patients was only about 50 times more than in healthy subjects (Figure 3A,B). Interestingly, levels of antagonist sgp130 increased in COVID-19 patients returning to those of the controls (312.12 ± 53.14 ng/mL vs. 308.24 ± 24.91 ng/mL) (Figure 3C).

### 3.2. Variants

VOCs have shown evidence of increased transmissibility and disease severity, as well as a significant reduction in neutralisation by antibodies generated during previous infection or vaccination. Therefore, we aimed to evaluate IL-6 and its soluble receptor complex levels in a subset of Delta variant COVID-19 infected patients.

**Figure 4:**
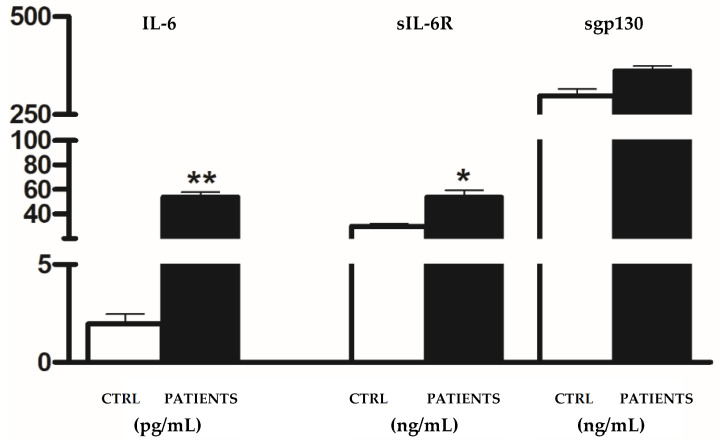
Analysis of IL-6 (pg/mL), sIL-6R (ng/mL) and sgp130 levels (ng/mL) in COVID-19 patients infected with Delta variant. Data are displayed as mean ± SD. Statistical analysis was performed with Mann–Whitney test with respect to Ctrl. * *p* < 0.05, ** *p* < 0.01.

Our results demonstrated that serum levels of IL-6 and sIL-6R remained significantly elevated in COVID-19 patients compared to healthy controls (54.05 ± 16.5 pg/mL vs. 1.95 ± 0.91 pg/mL, *p* < 0.01 and 53.83 ± 12.29 ng/mL vs. 30.51 ± 8.18 ng/mL, *p* < 0.05, respectively), whereas levels of the antagonist sgp130 returned to those of the controls (361.67 ± 45.14 ng/mL vs. 298 ± 25.02 ng/mL) (Figure 4).

## 4. Discussion

In Italy, like in many other countries, the COVID-19 pandemic has been characterised by multiple waves of infections. These waves, such as WAVE I, WAVE II, and WAVE III, represent distinct periods of increased transmission and varying impacts on public health and healthcare systems [19]. Monitoring the occurrence and dynamics of these waves is essential for implementing effective control measures and managing the pandemic.

Our study aimed to investigate the variability in serum levels of IL-6 and its soluble receptor complex among COVID-19 patients across different waves of the pandemic. Consistent with previous findings and the established role of IL-6 as a hallmark inflammatory marker in COVID-19 patients, we observed a significant increase in IL-6 levels in patients compared to healthy controls during all pandemic waves [20,21]. However, the magnitude of cytokine increases was notably lower in the second and third waves compared to the initial wave. Additionally, we observed (Figure 1B, Figure 2B, Figure 3B and Figure 4) an increase in the IL-6 agonist sIL-6R in all patients compared to healthy controls, indicating a sustained inflammatory response across all waves. Conversely, levels of the antagonist sgp130 were drastically reduced in patients during WAVE I but gradually returned to control levels by WAVE III. These findings underscore the central role of IL-6 in driving the cytokine storm associated with SARS-CoV-2 infection. Moreover, the imbalance between IL-6 agonistic and antagonistic molecules suggests a dynamic modulation of IL-6 levels associated with the severity of infection [22]. In particular, in WAVE I, we showed that great amounts of IL-6, about 400 times more in patients than healthy controls, were sustained by sIL-6R up-regulation, whereas sgp130 was not able to exert antagonist modulation. It is known that enhancements in serum concentrations of IL-6 and sIL-6R during infection lead to an increased agonistic trans-signalling mechanism [23]. At the same time, lower sgp130, which interacts with the IL-6/sIL-6R complex, leads to a decreased block in IL-6 trans-signalling, thus confirming the reduced antagonistic role of sgp130 during viral infection [24]. The contemporary changes in IL-6 and receptor concentration, as well as the consequent increase in signalling transduction, are closely associated with increased mortality and unfavourable outcomes in COVID-19 patients [25].

In a retrospective study, Gorham et al. assessed IL-6 concentrations in patients admitted to the ICU during the first wave of the pandemic, distinguishing between survivors and non-survivors. They observed a significant discrepancy in IL-6 levels, with non-survivors exhibiting considerably higher levels (720 pg/mL) compared to survivors (336 pg/mL). This led them to suggest repeated evaluation of IL-6 as a prognostic marker in critically ill patients with SARS-CoV-2-induced disease [26].

Subsequently, there was a proposal to employ IL-6 pathway blockade to manage cytokine release syndrome stemming from virus infection [27]. While initial data on anti-IL-6 agents, particularly tocilizumab, showed promise, early randomised trials yielded mixed results in terms of clinical benefit [28,29]. Larger trials such as RECOVERY and REMAP-CAP later established anti-IL-6 therapy, in combination with steroids, as a potential option for hypoxic patients exhibiting evidence of hyperinflammation [30]. Notably, results from critically ill patients where the cytokine storm had already commenced suggested that steroids may be more effective than tocilizumab in controlling the inflammatory reaction [31]. Furthermore, it is conceivable that the dysregulation of immunological mediators implicated in COVID-19, including IL-6, sIL-6R, and sgp130, may contribute to varied prognoses and responses to pharmacological interventions such as tocilizumab and steroids.

Several clinical investigations noted a less pronounced cytokine storm during the second wave of the pandemic compared to the first [29]. This could be attributed to better and faster treatments available to patients admitted to the ICU during the second wave. Our findings reflected a lower increase in IL-6 and sIL-6R levels among WAVE II patients compared to controls, while sgp130 levels exhibited a higher decrease relative to the control group. This suggests that patients during the second wave experienced less severe disease, leading to hospitalisation in a smaller percentage of cases. Consequently, the mortality rate was notably lower, possibly due to early COVID-19 diagnosis, improved management of infected patients, and better-prepared health systems, alongside preferential protection measures for older and higher-risk vulnerable individuals.

However, despite advancements, the second wave still resulted in excess mortality among patients over 70 years old, especially those with comorbidities. Additionally, the health consequences of COVID-19 persisted beyond acute infection, emphasising the need for ongoing surveillance of patients who have survived the acute phase of SARS-CoV-2 infection [31]. Towards the end of the second wave, despite high vaccination rates among the adult Italian population, a new surge in COVID-19 infections emerged, underscoring the ongoing challenges in managing the pandemic.

We found that levels of IL-6 and sIL-6R continued to be significantly increased in COVID-19 patients compared to healthy controls (63.15 ± 18.5 pg/mL vs. 1.91 ± 0.89 pg/mL and 75.21 ± 14.29 ng/mL vs. 26.51 ± 8.16 ng/mL, respectively). However, there was a substantial decrease in IL-6 values with respect to the previous waves. Interestingly, levels of antagonist sgp130 increased in COVID-19 patients, returning to those of the control (312.12 ± 53.14 ng/mL vs. 308.24 ± 24.91 ng/mL). Several clinical trials have demonstrated that in comparison with the first and second, patients in the third wave had fewer comorbidities and presented with moderate COVID-19 infection [32]. Indeed, the influence of age, gender, and comorbidities on the occurrence of severe COVID-19 was less marked in the third wave compared with the first two, and the interactions between age and comorbidities were less important. The changes in these clinical risk factors can be explained by a change in the exposed population as the virus spread in the population between the first waves and by the vaccination campaign [33]. Thus, it was expected that the generalisation of vaccination in a context where the virus and its recent variant forms continued to be very present in the general population could modify the profile of people severely affected by the disease. In particular, SARS-CoV-2-infected patients in WAVE III were less symptomatic, with intensive respiratory support being required in a limited number of cases, and an elevation of blood inflammatory markers (C-Reactive Protein and Neutrophil to Lymphocyte Ratio) was found in a lower proportion of cases.

The third wave of the pandemic was largely driven by the Delta variant of SARS-CoV-2 [34,35]. Our results showed that serum levels of IL-6 and sIL-6R continued to significantly increase in patients, whereas those of antagonist sgp130 returned to the levels of the controls. It is known that VOCs may impact the properties or behaviour of a virus, with consequences on virus transmissibility, disease severity or neutralisation mechanisms of antibodies generated during previous infection and vaccination. As such, our study provides additional support for the growing body of literature confirming the central role of IL-6 in COVID-19 and also in Delta-positive patients; however, further research is needed to understand the functions of agonist and antagonist molecules on disease changes relating to virus variants.

## 5. Conclusions

Our study provides valuable insights into the role of IL-6 in severe COVID-19 cases across different phases of the pandemic. The identification of the imbalance in its soluble receptor complex as a potential explanation for variations in infection severity underscores the importance of exploring these factors further. Given the current absence of specific treatments for COVID-19, the potential utility of sIL-6R and sgp130 as markers for disease progression, severity, and mortality risk is noteworthy. However, it is crucial to recognise that our findings represent a preliminary step, and further investigations are necessary to strengthen these conclusions. Continued research efforts will be essential for gaining a deeper understanding of the cytokine storm mechanism in COVID-19 infection and for refining approaches to disease management and prognosis assessment.

## Figures and Tables

**Table 1:** Demographic and clinical characteristics of enrolled patients.

	WAVE I	WAVE II	WAVE III
Patients	(N = 23)	(N = 104)	(N = 30)
Age	66.88 ± 14.48	68.72 ± 18.32	67.42 ± 14.86
Male (%)	18 (78.26%)	68 (65.38%)	23 (76.67%)
Diabetes (%)	9 (39.13%)	24 (23.12%)	4 (13.31%)
Hypertension (%)	18 (78.26%)	66 (63.46%)	13 (43.32%)
Chronic kidney disease (%)	8 (34.78%)	22 (21.15%)	7 (23.11%)
Died (%)	12 (52.17%)	48 (46.15%)	3 (10%)

## Data Availability

The data presented in this study are available on request from the corresponding author. The data are not publicly available due to the privacy of the clinical data.
